# Rapid and Comprehensive Analysis of 41 Harmful Substances in Multi-Matrix Products by Gas Chromatography–Mass Spectrometry Using Matrix-Matching Calibration Strategy

**DOI:** 10.3390/ma17102281

**Published:** 2024-05-11

**Authors:** Yue Wang, Dawei Xiong, Xiangke He, Lihua Yu, Guixiao Li, Tian Wang, Chongshu Liu, Zhongxian Liu, Zhi Li, Cuiling Gao

**Affiliations:** Shandong Institute for Product Quality Inspection, Jinan 250102, China; wangyue@sdqi.com.cn (Y.W.); xiongdawei@sdqi.com.cn (D.X.); hexiangke@sdqi.com.cn (X.H.); yulihua@sdqi.com.cn (L.Y.); liguixiao@sdqi.com.cn (G.L.); wangtian@sdqi.com.cn (T.W.); liuchongshu@sdqi.com.cn (C.L.); liuzhongxian@sdqi.com.cn (Z.L.); lizhi@sdqi.com.cn (Z.L.)

**Keywords:** phthalates, organophosphate flame retardants, polycyclic aromatic hydrocarbons, multi-matrix products, matrix effect, matrix-matching calibration strategy

## Abstract

Harmful substances in consumer goods pose serious hazards to human health and the environment. However, due to the vast variety of consumer goods and the complexity of their substrates, it is difficult to simultaneously detect multiple harmful substances in different materials. This paper presents a method for the simultaneous determination of 41 harmful substances comprising 17 phthalates (PAEs), 8 organophosphate flame retardants (OPFRs), and 16 polycyclic aromatic hydrocarbons (PAHs) in five types of products using the matrix-matching calibration strategy. The method employs an efficient ultrasonic extraction procedure using a mixture of dichloromethane and methylbenzene, followed by dissolution–precipitation and analysis through gas chromatography–mass spectrometry. Compared with previous experiments, we established a universal pretreatment method suitable for multi-matrix materials to simultaneously determine multiple harmful substances. To evaluate the effects of the matrix on the experimental results, we compared neat standard solutions and matrix-matching standard solutions. The results demonstrated that all compounds were successfully separated within 30 min with excellent separation efficiency. Additionally, the linear relationships of all analytes showed strong correlation coefficients (R^2^) of at least 0.995, ranging from 0.02 mg/L to 20 mg/L. The average recoveries of the target compounds (spiked at three concentration levels) were between 73.6 and 124.1%, with a relative standard deviation (n = 6) varying from 1.2% to 9.9%. Finally, we tested 40 different materials from consumer products and detected 16 harmful substances in 31 samples. Overall, this method is simple and accurate, and it can be used to simultaneously determine multiple types of hazardous substances in multi-matrix materials by minimizing matrix effects, making it an invaluable tool for ensuring product safety and protecting public health.

## 1. Introduction

Consumer goods are multifunctional substances that are closely related to human health. They have a wide variety of applications, such as toys, textiles, electronic and electrical products, and decorative materials. The consumer goods market has developed rapidly with the acceleration of global industrialization and urbanization. As chemicals are used in a large number of products, they can cause substantial harm to the health of consumers and the development of relevant industries [1]. In recent years, the concept of consumer product quality and safety has been developing with a focus on safety, health, and environmental protection, among which the chemical risks of consumer goods are attracting increasing attention from the international community. Strict safety regulations have been formulated by countries around the world, covering the entire lifecycle of chemicals from research and development to production, storage, use, and destruction. This has led to the establishment of a list of candidate chemicals, giving priority to high-concern chemicals and corresponding products. In this context, the study of harmful substances in consumer products is particularly important.

Phthalates (PAEs), organophosphate flame retardants (OPFRs), and polycyclic aromatic hydrocarbons (PAHs) are the most commonly restricted substances in consumer product regulations due to their ubiquity, persistence, and toxicity to humans and the environment [2,3]. The European regulation of chemicals (REACH), the Consumer Product Safety Improvement Act (CPSIA), and the Chemicals of High Concern to Children (CHCC) all specify prohibited or restricted hazardous chemicals, including phthalates (PAEs), organophosphate flame retardants (OPFRs), and polycyclic aromatic hydrocarbons (PAHs). Organophosphate flame retardants (OPFRs) and phthalates (PAEs) are widely used as additives to improve the plasticity and flame-retardant properties of textiles, furniture, electronics, coatings, plastics, and polyurethane foam because of their low price and high performance [4,5]. Phthalates (PAEs) have also been identified in personal perfume [6,7,8], and researchers have evaluated the potential genotoxicity of phthalate esters in perfumes using in vitro assays [9]. Polycyclic aromatic hydrocarbons (PAHs) are produced as a by-product of combustion and are usually introduced through the use of additives or the heating process of molding [10,11,12]. Phthalates (PAEs) have been discovered to have estrogenic properties [13,14,15,16], as well as significant reproductive toxicity [17,18,19,20], immunotoxicity [21,22], and carcinogenicity to organisms [18,23]. Due to the widespread application of PAEs and the diversity of transportation routes, PAE residues are found in many environments, such as air [24], water [25], and soil [26]. This poses a threat to ecological safety and human health. Organophosphate flame retardants (OPERs) have been associated with reproductive, developmental, brain and neuro-, bone development, cardiac development, endocrine, and metabolic toxicity [27,28,29,30,31]. All these chemicals exhibit carcinogenic, teratogenic, and mutagenic effects. Although the use of these substances is restricted, they are still detected in many consumer products as a result of both intentional and unintentional use [32]. Therefore, it is necessary to develop simple, fast, and accurate high-throughput detection technology for the simultaneous determination of harmful chemicals in consumer products. This would provide technical support to ensure the quality and safety of consumer products and improve the control measures of consumer products.

In order for consumer products to comply with the relevant regulations at home and abroad and avoid the resulting chemical harm to human beings, effective and reliable analytical methods are needed to identify harmful chemicals. The reported analytical methods mainly include gas chromatography (GC), gas chromatography–mass spectrometry (GC-MS), liquid chromatography (LC), and liquid chromatography–mass spectrometry (LC-MS). Gas chromatography–mass spectrometry (GC/MS) is a common method for testing plasticizers and polycyclic aromatic hydrocarbons in plastics. Some researchers have detected phthalates (PAEs) in plastics [18,21,33], personal perfume [6,7], and polymeric coatings [34] using GC/MS. In addition, PAEs in polyvinyl chloride (PVC) toys were also detected using ultra-high-performance liquid chromatography coupled with electronic spray mass spectrometry (UPLCMS/MS) [15]. Geiss, O. proposed a simple, fast, and cost-effective method for determining eight PAHs in rubber and plastic materials [35]. For substances with poor thermal stability and low volatility in materials, liquid chromatography can be applied for analysis, such as bisphenol A and phenol [36]. J.H. Sung et al. [37] determined the contents of 14 kinds of N-nitrosamines that migrated into artificial saliva in rubber soothers for children by liquid chromatography–tandem mass spectrometry, and the quantification was accurate. Generally, there are numerous pigments and other impurities present in the plastic matrix extract solution. The composition of the matrix can significantly impact the detection results. Gas chromatography–mass spectrometry offers high selectivity and sensitivity as well as strong resistance to matrix interference, and when combined with precipitation separation, it can potentially eliminate the purification process and facilitate rapid and accurate detection.

Polyvinyl chloride (PVC), acrylonitrile butadiene styrene (ABS), thermoplastic urethane (TPU), styrene–butadiene–styrene (SBS), and acrylic coatings are widely used in consumer products. Researchers have used several pretreatment methods for residue detection in different materials, such as microwave digestion [38], Soxhlet extraction [33,39,40], ultrasonic extraction [41,42,43], and ultrasonic-assisted solution matrix precipitation (USDP) [44]. Huang Lina et al. [33] detected phthalates in polyvinyl chloride (PVC) plastic toys using Soxhlet extraction–gas chromatography–mass spectrometry. Lucas Sternbauer et al. [38] determined the content of nucleating agents in plastics using microwave-assisted in situ derivation–extraction with gas chromatography and mass spectrometry, and selected tetrahydrofuran (THF) as the extraction solvent. The recovery rate of microwave extraction was 95.8~104.2%. Wang Qian et al. [45] used ultrasonic extraction combined with gas chromatography and mass spectrometry to separate and analyze the low-molecular-weight plasticizer in polyvinyl chloride (PVC) tubes. Chloroform was used as the extraction solvent, and the extraction efficiency was good. Shen Hao-Yu [46] used ultrasonic extraction combined with gas chromatography and mass spectrometry to screen and determine eight kinds of phthalates in plastic food products, and the extraction efficiency of n-hexane was superior. As a simple, cheap, and effective pretreatment method, dissolution–precipitation has been used to extract plasticizers, styrene and sensitizing aromatics from plastics [18,40,47]. All of these methods were proven to be effective for the extraction of different materials. However, most reports choose different pretreatment methods for different materials [48] and feature a complicated pretreatment process, long detection cycle, and solvent consumption. There are significant limitations in determining the presence of multiple harmful substances in multi-matrix products, in terms of both detection speed and scope, while the cost of detection is relatively high. In addition, most experiments do not consider the influence of the matrix effect when detecting harmful substances in plastics, which affects the accuracy of the results. Therefore, the selection of a universal pretreatment method for multi-matrix materials is particularly crucial to establish a high-throughput detection method.

In order to develop a simple, fast, and sensitive high-throughput detection technology for the simultaneous determination of harmful chemicals in consumer products, we developed a universal approach for the extraction and simultaneous detection of 41 analytes in multi-matrix materials. Through the use of ultrasonic extraction with two optimally selected solvents, followed by n-hexane precipitation, and utilizing gas chromatography and mass spectrometry, we achieved the simultaneous detection of phthalates (PAEs), organophosphate flame retardants (OPFRs), and polycyclic aromatic hydrocarbons (PAHs) in polyvinyl chloride (PVC), acrylonitrile butadiene styrene (ABS), thermoplastic urethane (TPU), styrene–butadiene–styrene (SBS), and acrylic coatings. Additionally, we thoroughly examined the matrix effect, linear range, recovery rate, and precision. The proposed method offers a rapid and simplified approach for the simultaneous detection of multiple harmful substances in multi-matrix samples of consumer products, thus contributing to the advancement of product safety and public health protection.

## 2. Materials and Methods

### 2.1. Chemicals and Reagents

The phthalate (PAE) mix 17 standard solution, the polycyclic aromatic hydrocarbon (PAH) mix 16 standard solution, and the standards of the 8 organophosphates considered in this work were obtained from ANPEL-TRACE Standard Technical Services (Shanghai, China). Specific reference material information is listed in Table 1.

The quality control samples of polyvinyl chloride (PVC), acrylonitrile butadiene styrene (ABS), thermoplastic urethane (TPU), styrene–butadiene–styrene (SBS), and acrylic coatings were obtained from Dongguan Dica Experimental Technology Co., Ltd., Dongguan, China. The standard values of analytes in each material are shown below: PVC: TCEP (31.9 mg/kg) and TCPP (48.0 mg/kg); TPU: TCEP (35.2 mg/kg) and TCPP (44.4 mg/kg); ABS: TCEP (27.4 mg/kg) and TCPP (41.3 mg/kg; SBS: NAP (1.98 mg/kg), ANY (6.56 mg/kg), ANA (10.2 mg/kg), FLU (9.23 mg/kg), PHE(12.5 mg/kg), ANT (13.4 mg/kg), FLT (14.8 mg/kg) and PYR (15.6 mg/kg); acrylic coating: NAP 23.1 mg/kg), ANY 6.76 mg/kg), ANA (6.74 mg/kg), FLU (7.41 mg/kg), PHE (8.66 mg/kg), ANT (7.83 mg/kg), FLT (9.09 mg/kg), and PYR (9.33 mg/kg). PVC: DIBP (970 mg/kg), DBP (885 mg/kg), BBP (1002 mg/kg), and DEHP (1056 mg/kg).

Ten typical organic solvents (methanol (chromatographic grade, purity > 99.9%), acetonitrile (chromatographic grade, purity > 99.9%), n-hexane (chromatographic grade, purity > 99.9%), toluene (chromatographic grade, purity > 99.9%), dichloromethane (chromatographic grade, purity > 99.9%)) were purchased from Thermo Fisher Technology Co., Ltd., Waltham, MA, USA); acetone (chromatographic grade, purity > 99%), ethyl acetate (chromatographic grade, purity > 99%), xylene (chromatographic grade, purity > 99%), and tetrahydrofuran (chromatographic grade, purity > 99%) were purchased from Shanghai Anpu Experimental Technology Co., Ltd., Shanghai, China); and N,N-dimethyl formamide (chromatographic grade, purity > 99%) was purchased from Sinopharm Group Chemical Reagent Co., Ltd., Shanghai, China.

A total of 40 samples were purchased from shopping malls and e-commerce platforms, including daily consumer goods such as raincoats, toys, balls, children’s learning chopsticks, socket protective covers, and decorative materials. They were composed of different materials such as polyvinyl chloride (PVC), acrylonitrile butadiene styrene (ABS), and thermoplastic urethane (TPU). 

### 2.2. Instrument and Analysis Conditions

An ISQ LT GC-MS/MS system (Thermo Fisher, Waltham, MA, USA) equipped with a TG-5 capillary column (30 m × 0.25 mm × 0.25 μm) was used. The samples were ultrasonicated using a KQ-500DB Ultrasonic Cleaner (Kunshan Ultrasonic Instrument Co., Ltd., Kunshan, China). The instrument used to crush the samples was a CryMill automatic freezing grinding instrument (Retsch, Haan, Germany). An electronic analytical balance was obtained from Sartorius Scientific Instruments (Beijing) Co., Ltd., Beijing, China. We used a nitrogen evaporator and a Nylon 66 filter membrane (0.22 μm), which were purchased from Shanghai Anpu Experimental Technology Co., Ltd., Shanghai, China.

The carrier gas was helium and the flow rate was 1.0 mL/min. The sample injection volume was 1.0 µL with unsplit stream sampling. Heating procedure: hold for 1 min at 60 °C and then heat to 210 °C at a rate of 12 °C/min and hold for 0 min; heat to 230 °C at a rate of 15 °C/min and hold for 0 min, and then heat to 250 °C at a rate of 3 °C/min and hold for 0 min; and finally, heat to 300 °C at a rate of 25 °C/min and hold for 7 min.

The samples were volatized with electron impact ionization and the voltage was 70 eV. The temperatures of the transmission and ion source were 290 and 280 °C; the quality scanning range was 50–500 *m*/*z*. Full scan mode was used, and the quantitative calculation was carried out using an external standard method. Other mass spectrum parameters are shown in Table 2.

### 2.3. Preparation of Standard Solution

Standard solution: Weigh 8 different organic phosphate esters and dissolve them with methanol to obtain individual stock solutions of each compound. Dilute them with methanol to prepare a 1000 mg/L mixed standard solution. Take 17 phthalate, 8 organophosphate ester, and 16 polycyclic aromatic hydrocarbon standard solutions; dissolve them in a fixed volume using a mixture of toluene, dichloromethane, and n-hexane (1:1:4, *V*/*V*/*V*); and prepare a series of standard working solutions at different concentrations of 0.02~20 mg/L.

Matrix-matching standard solution: Process the blank samples of polyvinyl chloride (PVC), acrylonitrile butadiene styrene (ABS), thermoplastic urethane (TPU), styrene–butadiene–styrene (SBS), and acrylic coatings according to the actual sample preparation method described in Section 2.4 to obtain the blank matrix extraction solution. Then, dilute the mixed standard stock solution to prepare different concentrations of the matrix-matching standard working solution. The different concentrations were prepared using the same process as the standard solution mentioned above.

### 2.4. Sample Pretreatment

**Figure 1:**
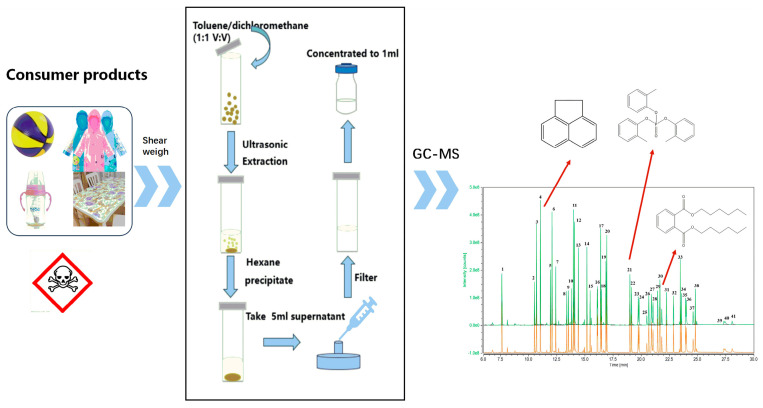
Schematic diagram of the analysis procedure. The chromatogram represents the peaks obtained with the neat standard solution (orange) and the matrix-matching standard solution (green).

As Figure 1 shows, the samples were cut into pieces smaller than 0.3 cm × 0.3 cm using scissors or a frozen grinder. Then, 0.3 g of the sample was weighed and transferred to a 40 mL glass extraction flask, followed by ultrasonic extraction with 10 mL of mixed solvent of toluene and dichloromethane (1:1, *V*/*V*) at 40 °C for 60 min. Additionally, 20 mL of slowly dripped n-hexane was added for dissolution and precipitation. The solution was continuously shaken and then left for ten minutes. Then, 5 mL of supernatant was filtered through a 0.22 μm organic filter membrane. After the nitrogen blowing process, it was concentrated to 1 mL and then analyzed according to the GC-MS conditions stated in Section 2.2. The chromatogram represents the peaks obtained with the neat standard solution (orange) and the matrix-matching standard solution (green).

## 3. Results

### 3.1. Chromatographic Mass Spectrometry

**Figure 2:**
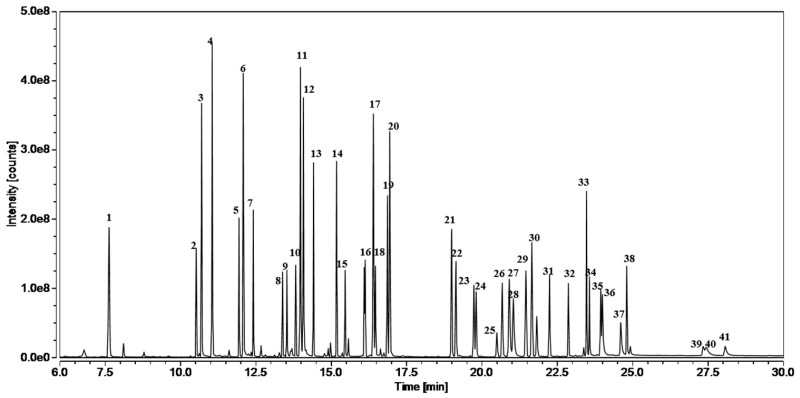
Total ion chromatogram of 41 harmful substances. The number codes are the same as those in Table 1.

The analytes included 17 phthalates (PAEs), 8 organophosphate flame retardants (OPFRs), and 16 polycyclic aromatic hydrocarbons (PAHs). The mixed standard solution of 10 mg/L obtained in Section 2.3 was analyzed according to the working conditions of the instrument stated in Section 2.2, resulting in the acquisition of a total ion chromatogram (TIC) for 41 compounds at an *m*/*z* range of 50~500 via GC-MS in full-scan mode, which is presented in Figure 2. We obtained the retention time of each analyte from the total ion chromatogram. As can be seen in Figure 2, the 41 harmful substances are well separated and have good peak shapes. The retention time, mass spectrometric parameters, CAS, and linear range of the substances are listed in Table 2. The order illustrated in Table 2 is based on the species of analytes. The “No.” in Table 2 corresponds to the number codes in Figure 2.

### 3.2. Optimization of Extraction Solvent and Precipitator

**Figure 3:**
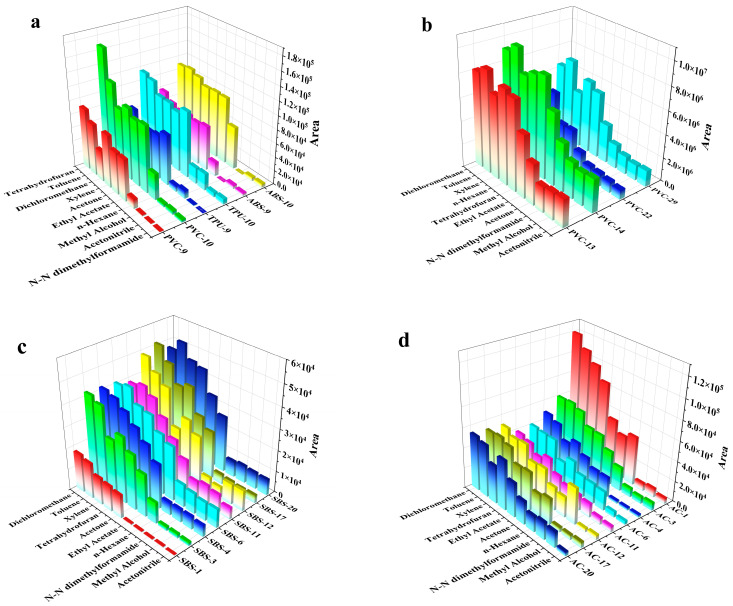
Extraction efficiency of 10 kinds of solvents for (**a**) OPFRs in PVC, ABS, and TPU; (**b**) PAEs in PVC; (**c**) PAHs in SBS; (**d**) PAHs in acrylic coatings (AC). The number codes next to the materials represent the analytes in Table 2.

Huang et al. determined the presence of prohibited phthalates in PVC plastic toys using Soxhlet extraction gas chromatography–mass spectrometry with dichloromethane as the extraction agent, and the detection limit, accuracy, and operation procedures were good [33]. Lucas et al. determined the content of nucleating agent in plastics using microwave-assisted in situ derivation extraction with gas chromatography and mass spectrometry. Tetrahydrofuran (THF) was selected as the extraction solvent, and the recovery rate was 95.8~104.2% [38]. These traditional extraction methods take a long time and require specialized equipment. To solve this problem, we used the ultrasonic extraction–solution–precipitation method to determine target analytes in plastics. In order to obtain suitable extraction solvents for different materials, we selected five quality control samples of polyvinyl chloride (PVC), acrylonitrile butadiene styrene (ABS), thermoplastic urethane (TPU), styrene–butadiene–styrene (SBS), and acrylate coatings. The analytes in these five materials are stated in Section 2.1. We investigated the extraction efficiency of analytes on quality control samples in methanol, acetone, n-hexane, ethyl acetate, toluene, xylene, dichloromethane, N, N-dimethyl formamide, tetrahydrofuran, and acetonitrile. Sample particles were pretreated in 10 solvents according to Section 2.4 and then analyzed according to the GC-MS conditions stated in Section 2.2. The extraction efficiency was compared using the peak area of the GCMS spectrum, and the extraction results are shown in Figure 3. Figure 3a shows a comparison of the extraction areas of organophosphate esters (OPFRs) in polyvinyl chloride (PVC), acrylonitrile butadiene styrene (ABS), and thermoplastic urethane (TPU). The numbers after the materials represent the analytes presented in Table 2; for example, PVC-9 represents TCEP in the PVC samples. Meanwhile, Figure 3b–d show a comparison of the extraction areas of phthalic plasticizers (PAEs) in PVC, PAHs in thermoplastic urethane (TPU), and PAHs in acrylate coatings (AC). As shown in the figures, the most effective solvents for the five materials were toluene and dichloromethane under the same extraction conditions. Considering the different chemical properties of various materials, a mixture of dichloromethane and toluene (1:1, *V*/*V*) with different polarity was selected as the extraction solvent. This approach allowed us to effectively extract compounds from the five types of materials, which is verified in the following results. 

**Figure 4:**
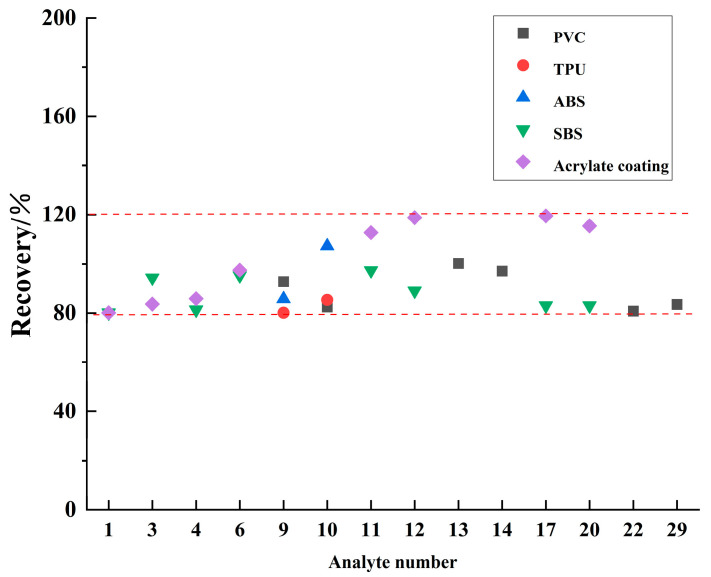
Recovery of organophosphate flame retardants (OPFRs) in polyvinyl chloride (PVC), acrylonitrile butadiene styrene (ABS), and thermoplastic urethane (TPU); phthalates (PAEs) in polyvinyl chloride (PVC); and polycyclic aromatic hydrocarbons (PAHs) in styrene–butadiene–styrene (SBS) and acrylic coating. The number codes represent the analytes in Table 2. The red dotted lines represent the recovery rates of 80.1% and 119.5%.

To verify the effective extraction of the mixed solution, we pretreated five quality control samples of polyvinyl chloride (PVC), acrylonitrile butadiene styrene (ABS), thermoplastic urethane (TPU), styrene–butadiene–styrene (SBS), and acrylate coating. Sample particles were prepared in dichloromethane and toluene (1:1, *V*/*V*) according to the method in Section 2.4 and then analyzed according to the GC-MS conditions stated in Section 2.2. The analytes studied in the five quality control samples were the same as in Section 2.1. The recovery rate was then measured. As Figure 4 reveals, the recovery rate of harmful substances in these five materials ranged from 80.1% to 119.5%, indicating a satisfactory recovery rate. The experiment demonstrated that the mixed solution of dichloromethane and toluene (1:1, *V*/*V*) can effectively extract these materials with a satisfactory recovery rate. 

Dissolution–precipitation is a simple, cheap, and effective pretreatment method that uses organic solvent extraction and polymer precipitation to transfer a target substance to a supernatant. Gimeno Pascal used ethanol as a precipitant in his experiments to determine plasticizers in PVC medical devices through GC-MS [39]. Mahmoud Alawi used n-hexane as a precipitant to extract plasticizers from plastics [18], and Lv, Q used methanol as a precipitant to determine 48 fragrance allergens in toys using GC with ion trap MS/MS [47]. Due to the presence of polymer molecules during the precipitation process, there is a likelihood of adsorbing plasticizers and other harmful substances, which can lead to lower determination results. In this study, methanol and n-hexane were utilized to precipitate five distinct extracted materials. It was observed that the materials exhibited diverse reactions to the precipitators. Specifically, flocculation occurred readily when methanol was employed, whereas n-hexane facilitated the formation of uniform and fine precipitates. Although both solutions were clarified after filtration, the n-hexane solution remained unchanged after nitrogen blowing concentration, while the methanol solution became turbid, necessitating the replacement of the clarified solution with a fresh solvent. Ultimately, n-hexane was selected as the preferred precipitation solvent in this experiment. The findings of this study demonstrate that the adsorption effect of the precipitant on harmful substances can be negligible.

### 3.3. Investigation of Matrix Effect

**Figure 5:**
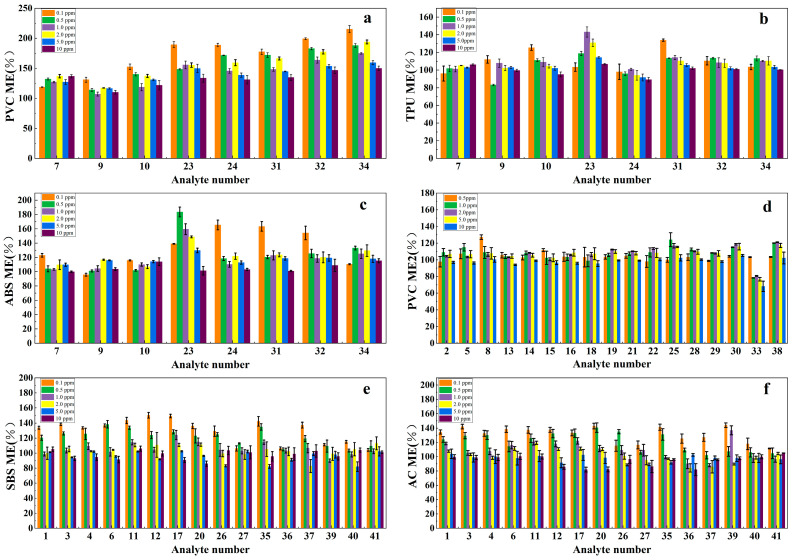
Matrix effects of organophosphate flame retardants (OPFRs) in PVC (**a**), TPU (**b**), ABS (**c**); phthalates (PAEs) in PVC (**d**); and polycyclic aromatic hydrocarbons (PAHs) in SBS (**e**) and acrylic coating (AC) (**f**) at different concentrations. The number codes are the same as those in Table 1.

The composition of plastic matrices is highly complex, and some investigations utilize an SPE column for purification techniques [47], which adds extra pretreatment steps and increases the detection cost. Conversely, some studies opt not to purify the solution and inject the compound directly. If the matrix effect is not factored in, the sample’s test condition may diverge from the calibration curve’s standard sample, resulting in potential test discrepancies. The ME is defined as “the combined effect of all components of the sample other than the analyte on the measurement of the quantity” by the International Union of Pure and Applied Chemistry (IUPAC). The ME is usually expressed as the ratio of the peak area of an analyte standard before and after extraction. A value of 100% indicates that no MEs are observed. A value >100% represents an enhancement of the signal, while a value <100% is indicative of a suppression effect. This study delved into the influence of matrix effects (MEs) on experimental outcomes. Under the same conditions, we prepared different concentrations of gradient standard solutions with toluene–dichloromethane–n-hexane (1:1:4, *V*/*V*/*V*) solvent and matrix-matching solution, respectively. The separation and response values of the target substance at the same concentration were compared with the results obtained from the GC/MS. The chromatographic analysis of two standard solutions revealed that the different solvents had no effect on the order or the time of the harmful substances. We opted to compare the matrix effects of toxic and harmful substances in distinct materials by selecting eight organophosphate esters in PVC, ABS, and TPU and 17 phthalic plasticizers in PVC. The response value of the mixed solvent to the plasticizer was lower than that of the blank matrix extract solvent. As indicated in Figure 5, various harmful substances in PVC, ABS, and TPU generally display a matrix enhancement effect, with the matrix effect varying at different concentrations. Notably, the matrix effect is more pronounced at lower concentrations and shows a weakening trend with the increase in concentration [49]. Even within the same substrate, the matrix effects of different harmful substances can vary significantly, which is attributed to the polarity of the harmful substances. Previous studies have demonstrated that the greater the polarity, the more prominent the matrix effect [50,51]. Therefore, in actual sample testing, the standard solution should be mixed with a blank matrix-matching solution to compensate for any discrepancies in the response value of harmful substances in the standard solution and the sample solution to the same extent.

### 3.4. Method Validation

The method was validated according to ICH Q2 guidance. The optimized methods were subjected to the following validation parameters: limit of detection (LOD), limit of quantitation (LOQ), linear range, linearity (R^2^), precision, and accuracy. The calibration curves were generated in the range of 0.4~200 mg/kg. To plot the standard working curve, the peak area of the chromatogram was taken as the vertical coordinate, while the corresponding content of each substance was taken as the horizontal coordinate. The results revealed that all substances exhibited good linear relationships within their respective linear ranges, with correlation coefficients (R^2^) greater than 0.995. The standard solutions of the 17 PAEs, eight OPFRs, and 16 PAHs prepared from the blank matrix extract were determined from a low concentration to a high concentration. The LODs were determined as the concentration of analyte that resulted in a signal-to-noise ratio (S/N) of 3:1. The LOQs were defined as the concentration of analyte that produced an S/N of 10:1. The matrix-matched LODs of the instruments were 0.01~0.02 mg/L (eight OPFRs), 0.15~0.26 mg/L (17 PAEs), and 0.01~0.05 mg/L (16 PAHs). The LODs of the method for each substance were 0.1~0.5 mg/kg (eight OPFRs), 3.0~5.3 mg/kg (17 PAEs), and 0.2~1.0 mg/kg (16 PAHs). The LOQs of the instruments were 0.03~5.57 mg/L (eight OPFRs), 0.50~0.87 mg/L (17 PAEs), and 0.03~0.17 mg/L (16 PAHs). The LOQs of the method were 0.33~1.67 mg/kg (eight OPFRs), 10~17.67 mg/kg (17 PAEs), and 0.67~3.3 mg/kg (16 PAHs). In previous research, Lina Huang determined the presence of phthalates in plastics; the quantitative LOD of the instruments was 0.17–1.86 mg/L and the LODs of the method were 5–15 mg/kg (DBP, BBP, DEHP, and DNOP) and 50 mg/kg (DINP and DIDP) [33]. Our LOD and LOQ values are similar to those in previous studies [18,39].

**Figure 6:**
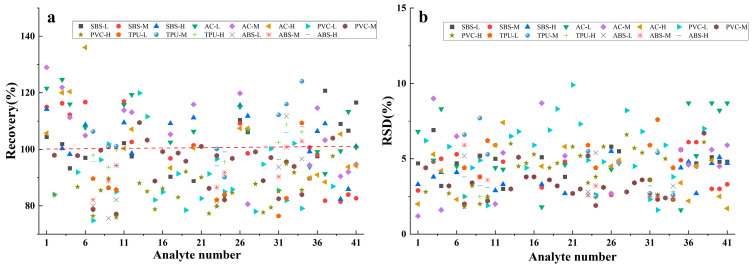
Recoveries (**a**) and RSD (**b**) for 41 substances in 5 kinds of materials (PVC, TPU, ABS, SBS, and acrylic coatings). The number codes are the same as those in Table 1. The red dotted line represent the recovery of 100%.

A mixed standard solution with three concentration levels of high, medium, and low was added to the blank matrix sample, and the standard recovery and relative standard deviation (RSD) of the measured value were calculated six times according to the experimental method. The results are shown in Figure 6 (L—low concentration; M—medium concentration; H—high concentration; AC represents the acrylic coating). As can be seen from Figure 6, the recoveries of organic phosphate ester in PVC ranged from 74.8% to 119.9%, with RSDs (n = 6) varying from 1.6% to 4.9%. For the ABS samples, the recoveries of organic phosphate ester ranged from 80.9% to 111.7%, with RSDs ranging from 2.4% to 5.9%. The recoveries of organophosphate in TPU were 76.4–124.1%, with RSDs of 2.5–7.7%. The recoveries of phthalic plasticizers in PVC ranged from 77.7% to 119.9%, with RSDs of 2.8–9.9%. The recoveries of PAHs in SBS were 76.5–97.3%, with RSDs of 2.6–6.9%. The recoveries of PAHs were 73.6–119.5%, with RSDs of 1.2–9.0%. The results indicate that the method is accurate and repeatable for the detection of various hazardous substances.

### 3.5. Analysis of Commercial Products

**Figure 7:**
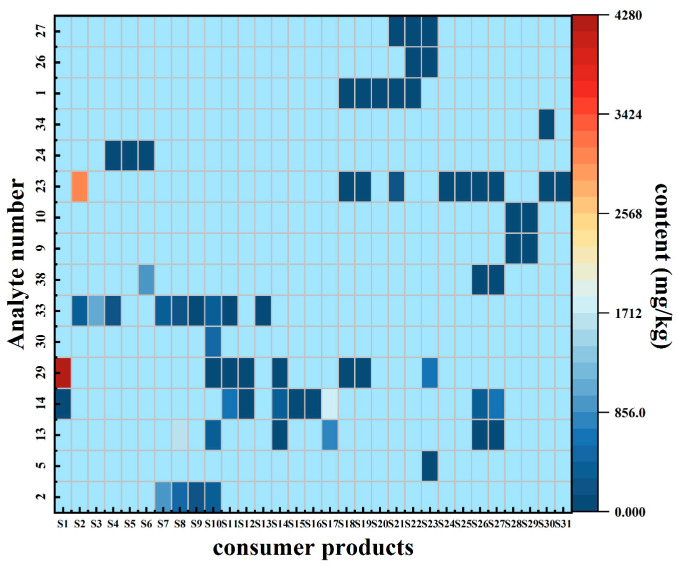
Results of consumer products in which at least 1 of the 41 analytes was found (S1: Sample 1). The number codes are the same as those in Table 1.

The proposed method was applied for the analysis of 20 PVC samples, 10 ABS samples, and 10 TPU samples, which were collected from shopping malls and e-commerce platforms, including daily consumer goods such as raincoats, toys, balls, children’s learning chopsticks, socket protective covers, and decorative materials (S1–S17: PVC materials; S18–S27: TPU materials; S28–S31: ABS materials). As shown in Figure 7, phthalic plasticizers and organophosphate esters were detected in 17 PVC samples. Eight analytes were detected: DMP (number 2), DIBP (number 13), DBP (number 14), DEHP (number 29), DPHP (number 30), DNOP (number 33), DNP (number 38), and TBEP (number 23). Among them, the hazardous substances with the highest detection rate were DBP (number 14) and DNOP (number 33). Additionally, DIBP, DBP, and DEHP exceeded the limit value of 1000 mg/kg stipulated in the EU toy standard and REACH regulation in four PVC samples. Organophosphate ester, phthalic plasticizers, and polycyclic aromatic hydrocarbons were detected in 10 TPU samples, and a total of nine substances were detected: DEP (number 5), DEHP (number 29), DIBP (number 13), DBP (number 14), DNP (number 38), tris(2-butoxyethyl) phosphate (number 23), NAP (number 1), BaA (number 26), and CHR (number 27). Among the 10 ABS samples tested, 5 samples contained at least one harmful substance: TCEP (number 9), TCPP (number 10), TBEP (number 23), and tris(4-methylphenyl) phosphate (number 34).

## 4. Conclusions

In this study, we presented a versatile and fast determination method based on ultrasonic extraction and dissolution–precipitation combined with GC-MS analysis using a matrix-matching calibration strategy for detecting multiple harmful substances in multi-matrix products. Compared with the traditional extraction method, the proposed method is simpler, faster, and more efficient. We systematically studied the matrix effects, extraction efficiency, linearity, limits of detection (LODs), precision, and recovery and then applied the method to the analysis of commercial samples. The results can serve as a reference for the pretreatment of complex samples. The application of the method to commercial samples revealed the presence of various target chemicals that may pose a threat to human health. This method can not only detect multiple harmful substances simultaneously but also provides a good solution for the detection of products with mixed materials, such as multi-layer co-extruded packaging materials. Further research is needed on the chemical risk assessment of consumer products.

## Figures and Tables

**Table 1:** Information of reference compounds.

Class	Compound Name	Abbreviations	Chemical Formula	CAS	Purity or Concentration
Organophosphate flame retardants (OPFRs)	Tributylphosphate	TBP	C_12_H_27_O_4_P	126-73-8	99.3%
	Tris(2-chloroethyl) phosphate	TCEP	C_6_H_12_C_l3_O_4_P	115-96-8	96.3%
	Tris(2-chloroisopropyl) phosphate	TCPP	C_9_H_18_C_l3_O_4_P	13674-84-5	98.2%
	Tris(2-butoxyethyl) phosphate	TBEP	C_18_H_39_O_7_P	78-51-3	95.2%
	Triphenyl phosphate	TPP	C_18_H_15_O_4_P	115-86-6	99.9%
	Tri-o-cresyl phosphate	/	C_21_H_21_O_4_P	78-30-8	99.1%
	Tris-m-cresyl phosphate	/	C_21_H_21_O_4_P	563-04-2	97.7%
	Tri-p-cresyl phosphate	/	C_21_H_21_O_4_P	78-32-0	99.4%
Polycyclic aromatic hydrocarbon (PAH) mix 16 standard solution	Naphthalene	NAP	C_10_H_8_	91-20-3	2000 mg/L in benzene/dichloromethane (1:1)
	Acenaphthylene	ACY	C_12_H_8_	208-96-8	
	Acenaphthen	ACE	C_12_H_10_	83-32-9	
	Fluorene	FLU	C_13_H_10_	86-73-7	
	Phenanthrene	PHE	C_14_H_10_	85-01-8	
	Anthracene	ANT	C_14_H_10_	120-12-7	
	Fluoranthene	FLA	C_16_H_10_	206-44-0	
	Pyrene	PYR	C_16_H_10_	129-00-0	
	1,2-Benzanthracene	BaA	C_18_H_12_	56-55-3	
	Chrysene	CHR	C_18_H_12_	218-01-9	
	Benzo[b]fluoranthene	BbF	C_20_H_12_	205-99-2	
	Benzo[k]fluoranthrene	BkF	C_20_H_12_	207-08-9	
	Benzo[a]pyrene	BaP	C_20_H_12_	50-32-8	
	Indeno(1,2,3-cd)pyrene	IcdP	C_22_H_12_	193-39-5	
	Dibenz[a,h]anthracene	DahA	C_22_H_14_	53-70-3	
	Benzo[ghi]perylene	BghiP	C_22_H_12_	191-24-2	
Phthalate (PAE) mix 17 standard solution	Dimethyl phthalate	DMP	C_10_H_10_O_4_	131-11-3	1000 mg/L in n-hexane
	Diethyl phthalate	DEP	C_12_H_14_O_4_	84-66-2	
	Diallyl phthalate	DAP	C_14_H_14_O_4_	131-17-9	
	Diisobutyl phthalate	DIBP	C_16_H_22_O_4_	84-69-5	
	Dibutyl phthalate	DBP	C_16_H_22_O_4_	84-74-2	
	Bis(2-methoxyethyl) phthalate	DMEP	C_14_H_18_O_6_	117-82-8	
	phthalic,biis-4-methyl-2-pentylester	/	C_20_H_30_O_4_	84-63-9	
	Bis(2-ethoxyethyl) phthalate	DEEP	C_16_H_22_O_6_	605-54-9	
	Dipentyl phthalate	DPP	C_18_H_26_O_4_	131-18-0	
	Di-n-hexyl phthalate	DHXP	C_20_H_30_O_4_	84-75-3	
	Butyl benzyl phthalate	BBP	C_19_H_20_O_4_	85-68-7	
	Bis(2-nbutoxyethyl)phthalate	DBEP	C_20_H_30_O_6_	117-83-9	
	Dicyclohexyl phthalate	DCHP	C_20_H_26_O_4_	84-61-7	
	Bis(2-ethylhexyl) phthalate	DEHP	C_24_H_38_O_4_	117-81-7	
	Diphenyl phthalate	DPhP	C_20_H_14_O_4_	84-62-8	
	Di-n-octyl phthalate	DNOP	C_24_H_38_O_4_	117-84-0	
	Dinonyl phthalate	DNP	C_26_H_42_O_4_	84-76-4	

**Table 2:** Retention time, mass spectrometric parameters, and linear range of 41 harmful substances.

No.	Compounds	CAS	Retention Time /min	Characteristic Fragment Ion (*m*/*z*)			Linear Range (mg/kg)				
							PVC	ABS	TPU	SBS	Acrylate Coating
**Organophosphate flame retardants (OPFRs)**											
7	Tributylphosphate (TBP)	126-73-8	12.415	99	155	57	2.0–200	2.0–200	2.0–200	2.0–200	2.0–200
9	Tris(2-chloroethyl) phosphate (TCEP)	115-96-8	13.534	63	249	143	2.0–200	2.0–200	2.0–200	2.0–200	2.0–200
10	Tris(2-chloroisopropyl) phosphate (TCPP)	13674-84-5	13.823	125	99	157	2.0–200	2.0–200	2.0–200	2.0–200	2.0–200
23	Tris(2-butoxyethyl) phosphate (TBEP)	78-51-3	19.748	57	56	85	2.0–200	2.0–200	2.0–200	2.0–200	2.0–200
24	Triphenyl phosphate (TPP)	115-86-6	19.829	326	325	77	2.0–200	2.0–200	2.0–200	2.0–200	2.0–200
31	Tri-2-cresyl phosphate	78-30-8	22.251	165	91	179	2.0–200	2.0–200	2.0–200	2.0–200	2.0–200
32	Tris-3-cresyl phosphate	563-04-2	22.867	368	367	91	2.0–200	2.0–200	2.0–200	2.0–200	2.0–200
34	Tri-4-cresyl phosphate	78-32-0	23.564	368	367	107	2.0–200	2.0–200	2.0–200	2.0–200	2.0–200
**Polycyclic aromatic hydrocarbons (PAHs)**											
1	Naphthalene (NAP)	91-20-3	7.619	128	129	127	0.4–200	0.4–200	0.4–200	0.4–200	0.4–200
3	Acenaphthylene (ACY)	208-96-8	10.697	152	153	151	0.4–200	0.4–200	0.4–200	0.4–200	0.4–200
4	Acenaphthene (ACE)	83-32-9	11.051	154	153	152	0.4–200	0.4–200	0.4–200	0.4–200	0.4–200
6	Fluorene (FLU)	86-73-7	12.078	166	165	167	0.4–200	0.4–200	0.4–200	0.4–200	0.4–200
11	Phenanthrene (PHE)	85-01-8	13.976	178	179	176	0.4–200	0.4–200	0.4–200	0.4–200	0.4–200
12	Anthracene (ANT)	120-12-7	14.075	178	179	176	0.4–200	0.4–200	0.4–200	0.4–200	0.4–200
17	Fluoranthene (FLA)	206-44-0	16.394	202	203	200	0.4–200	0.4–200	0.4–200	0.4–200	0.4–200
20	Pyrene (PYR)	129-00-0	16.935	202	203	200	0.4–200	0.4–200	0.4–200	0.4–200	0.4–200
26	1,2-Benzanthracene (BaA)	56-55-3	20.914	228	229	226	2.0–200	2.0–200	2.0–200	2.0–200	2.0–200
27	Chrysene (CHR)	218-01-9	21.054	228	226	113	2.0–200	2.0–200	2.0–200	2.0–200	2.0–200
35	Benzo[b]fluoranthene (BbF)	205-99-2	23.938	252	253	250	2.0–200	2.0–200	2.0–200	2.0–200	2.0–200
36	Benzo[k]fluoranthrene (BkF)	207-08-9	23.992	252	253	250	2.0–200	2.0–200	2.0–200	2.0–200	2.0–200
37	Benzo[a]pyrene (BaP)	50-32-8	24.598	252	253	250	2.0–200	2.0–200	2.0–200	2.0–200	2.0–200
39	Indeno(1,2,3-cd)pyrene (IcdP)	193-39-5	27.332	276	138	277	2.0–200	2.0–200	2.0–200	2.0–200	2.0–200
40	Dibenz[a,h]anthracene (DahA)	53-70-3	27.438	278	279	276	2.0–200	2.0–200	2.0–200	2.0–200	2.0–200
41	Benzo[ghi]perylene (BghiP)	191-24-2	28.060	276	277	138	2.0–200	2.0–200	2.0–200	2.0–200	2.0–200
**Phthalates (PAEs)**											
2	Dimethyl phthalate (DMP)	131-11-3	10.541	163	77	76	10–400	10–400	10–400	10–400	10–400
5	Diethyl phthalate (DEP)	84-66-2	11.963	149	177	150	10–400	10–400	10–400	10–400	10–400
8	Diallyl phthalate (DAP)	131-17-9	13.408	41	149	189	10–400	10–400	10–400	10–400	10–400
13	Diisobutyl phthalate (DIBP)	84-69-5	14.435	149	57	150	10–400	10–400	10–400	10–400	10–400
14	Dibutyl phthalate (DBP)	84-74-2	15.201	149	150	41	10–400	10–400	10–400	10–400	10–400
15	Bis(2-methoxyethyl) phthalate (DMEP)	117-82-8	15.486	59	58	149	10–400	10–400	10–400	10–400	10–400
16	phthalic,biis-4-methyl-2-pentylester	84-63-9	16.153	149	167	85	10–400	10–400	10–400	10–400	10–400
18	Bis(2-ethoxyethyl) phthalate (DEEP)	605-54-9	16.490	72	73	45	10–400	10–400	10–400	10–400	10–400
19	Dipentyl phthalate (DPP)	131-18-0	16.898	149	150	237	10–400	10–400	10–400	10–400	10–400
21	Di-n-hexyl phthalate (DHXP)	84-75-3	19.037	149	150	251	10–400	10–400	10–400	10–400	10–400
22	Butyl benzyl phthalate (BBP)	85-68-7	19.177	149	91	206	10–400	10–400	10–400	10–400	10–400
25	Bis(2-nbutoxyethyl)phthalate (DBEP)	117-83-9	20.724	149	57	101	10–400	10–400	10–400	10–400	10–400
28	Dicyclohexyl phthalate (DCHP)	84-61-7	21.510	149	167	150	10–400	10–400	10–400	10–400	10–400
29	Bis(2-ethylhexyl) phthalate (DEHP)	117-81-7	21.707	149	167	57	10–400	10–400	10–400	10–400	10–400
30	Diphenyl phthalate (DPhP)	84-62-8	21.857	225	77	226	10–400	10–400	10–400	10–400	10–400
33	Di-n-octyl phthalate (DNOP)	117-84-0	23.493	149	279	43	10–400	10–400	10–400	10–400	10–400
38	Dinonyl phthalate (DNP)	84-76-4	24.833	149	293	150	10–400	10–400	10–400	10–400	10–400

## Data Availability

Data are contained within the article.

## References

[1] Yang Y., Sun Z., Liu X., Jia W., Wu J. (2022). Optimal Decisions on Harmful Chemical Limits in Consumer Goods within an Acceptable Risk Level. Processes.

[2] Greaves A.K., Letcher R.J. (2017). A Review of Organophosphate Esters in the Environment from Biological Effects to Distribution and Fate. Bull. Environ. Contam. Toxicol..

[3] Wang Y., Zhang Z., Xu Y., Rodgers T.F.M., Ablimit M., Li J., Tan F. (2023). Identifying the Contributions of Root and Foliage Gaseous/Particle Uptakes to Indoor Plants for Phthalates, Opfrs and Pahs. Sci. Total Environ..

[4] Zhang Q., Wang Y., Zhang C., Yao Y., Wang L., Sun H. (2022). A Review of Organophosphate Esters in Soil: Implications for the Potential Source, Transfer, and Transformation Mechanism. Environ. Res..

[5] Sun J., Pan L., Tsang D.C.W., Zhan Y., Zhu L., Li X. (2018). Organic Contamination and Remediation in the Agricultural Soils of China: A Critical Review. Sci. Total Environ..

[6] Orecchio S., Indelicato R., Barreca S. (2015). Determination of Selected Phthalates by Gas Chromatography-Mass Spectrometry in Personal Perfumes. J. Toxicol. Environ. Health Part A.

[7] Huang P., Liao K., Chang J., Chan S., Lee C. (2018). Characterization of Phthalates Exposure and Risk for Cosmetics and Perfume Sales Clerks. Environ. Pollut..

[8] Al-Saleh I., Elkhatib R. (2016). Screening of Phthalate Esters in 47 Branded Perfumes. Environ. Sci. Pollut. Res..

[9] Al-Saleh I., Al-Rajudi T., Al-Qudaihi G., Manogaran P. (2017). Evaluating the Potential Genotoxicity of Phthalates Esters (Paes) in Perfumes Using in Vitro Assays. Environ. Sci. Pollut. Res..

[10] Oliveira M., Slezakova K., Delerue-Matos C., Pereira M.C., Morais S. (2019). Children Environmental Exposure to Particulate Matter and Polycyclic Aromatic Hydrocarbons and Biomonitoring in School Environments: A Review on Indoor and Outdoor Exposure Levels, Major Sources and Health Impacts. Environ. Int..

[11] Dat N., Chang M.B. (2017). Review on Characteristics of Pahs in Atmosphere, Anthropogenic Sources and Control Technologies. Sci. Total Environ..

[12] Abdel-Shafy H.I., Mansour M.S.M. (2016). A Review on Polycyclic Aromatic Hydrocarbons: Source, Environmental Impact, Effect On Human Health and Remediation. Egypt. J. Pet..

[13] Niino T., Ishibashi T., Itho T., Sakai S., Ishiwata H., Yamada T., Onodera S. (2002). Simultaneous Determination of Phthalate Di- And Monoesters in Poly(Vinylchloride) Products and Human Saliva by Gas Chromatography-Mass Spectrometry. J. Chromatogr. B.

[14] Ashworth M., Chappell A., Ashmore E., Fowles J. (2018). Analysis and Assessment of Exposure to Selected Phthalates Found in Children’S Toys in Christchurch, New Zealand. Int. J. Environ. Res. Public Health.

[15] Chen B., Zhang L. (2013). An Easy and Sensitive Analytical Method of Determination of Phthalate Esters in Children’s Toys by Uplcms/Ms. Polym. Test..

[16] Korfali S.I., Sabra R., Jurdi M., Taleb R.I. (2013). Assessment of Toxic Metals and Phthalates in Children’S Toys and Clays. Arch. Environ. Contam. Toxicol..

[17] Wittassek M., Koch H.M., Angerer J., Brüning T. (2011). Assessing Exposure to Phthalates–the Human Biomonitoring Approach. Mol. Nutr. Food Res..

[18] Al-Natsheh M., Alawi M., Fayyad M., Tarawneh I. (2015). Simultaneous Gc–Ms Determination of Eight Phthalates in Total and Migrated Portions of Plasticized Polymeric Toys and Childcare Articles. J. Chromatogr. B.

[19] Stringer R., Labunska I., Santillo D., Johnston P., Siddorn J., Stephenson A. (2000). Concentrations of Phthalate Esters and Identification of Other Additives in Pvc Children’s Toys. Environ. Sci. Pollut. Res..

[20] Ionas A.C., Dirtu A.C., Anthonissen T., Neels H., Covaci A. (2014). Downsides of the Recycling Process: Harmful Organic Chemicals in Children’s Toys. Environ. Int..

[21] Negev M., Berman T., Reicher S., Sadeh M., Ardi R., Shammai Y. (2018). Concentrations of Trace Metals, Phthalates, Bisphenol a and Flame-Retardants in Toys and Other Children’s Products in Israel. Chemosphere.

[22] Jo S., Lee M., Kim K., Kumar P. (2018). Characterization and Flux Assessment of Airborne Phthalates Released from Polyvinyl Chloride Consumer Goods. Environ. Res..

[23] Ting K., Gill M., Garbin O. (2009). Gc/Ms Screening Method for Phthalate Esters in Children’s Toys. J. Aoac Int..

[24] Xie Z., Selzer J., Ebinghaus R., Caba A., Ruck W. (2006). Development and Validation of a Method for the Determination of Trace Alkylphenols and Phthalates in the Atmosphere. Anal. Chim. Acta.

[25] Gao D., Li Z., Wen Z., Ren N. (2014). Occurrence and Fate of Phthalate Esters in Full-Scale Domestic Wastewater Treatment Plants and their Impact on Receiving Waters Along the Songhua River in China. Chemosphere.

[26] Gao D., Li Z., Wang H., Liang H. (2018). An Overview of Phthalate Acid Ester Pollution in China Over the Last Decade: Environmental Occurrence and Human Exposure. Sci. Total Environ..

[27] McGee S.P., Konstantinov A., Stapleton H.M., Volz D.C. (2013). Aryl Phosphate Esters within a Major Pentabde Replacement Product Induce Cardiotoxicity in Developing Zebrafish Embryos: Potential Role of the Aryl Hydrocarbon Receptor. Toxicol. Sci..

[28] Han Z., Wang Q., Fu J., Chen H., Zhao Y., Zhou B., Gong Z., Wei S., Li J., Liu H. (2014). Multiple Bio-Analytical Methods to Reveal Possible Molecular Mechanisms of Developmental Toxicity in Zebrafish Embryos/Larvae Exposed to Tris(2-Butoxyethyl) Phosphate. Aquat. Toxicol..

[29] Schang G., Robaire B., Hales B.F. (2016). Organophosphate Flame Retardants Act as Endocrine-Disrupting Chemicals in Ma-10 Mouse Tumor Leydig Cells. Toxicol. Sci..

[30] Blum A., Behl M., Birnbaum L.S., Diamond M.L., Phillips A., Singla V., Sipes N.S., Stapleton H.M., Venier M. (2019). Organophosphate Ester Flame Retardants: Are they a Regrettable Substitution for Polybrominated Diphenyl Ethers?. Environ. Sci. Technol. Lett..

[31] Wiersielis K.R., Adams S., Yasrebi A., Conde K., Roepke T.A. (2020). Maternal Exposure to Organophosphate Flame Retardants Alters Locomotor and Anxiety-Like Behavior in Male and Female Adult Offspring. Horm. Behav..

[32] Goyak K.O., Kung M.H., Chen M., Aldous K.K., Freeman J.J. (2016). Development of a Screening Tool to Prioritize Testing for the Carcinogenic Hazard of Residual Aromatic Extracts and Related Petroleum Streams. Toxicol. Lett..

[33] Huang L., Liu Z., Yi L., Liu C., Yang D. (2011). Determination of the Banned Phthalates in Pvc Plastic of Toys by the Soxhlet Extraction-Gas Chromatography/Mass Spectrometry Method. Int. J. Chem..

[34] Vázquez-Loureiro P., Lestido-Cardama A., Sendón R., López-Hernández J., Paseiro-Losada P., Rodríguez-Bernaldo De Quirós A. (2021). Identification of Volatile and Semi-Volatile Compounds in Polymeric Coatings Used in Metal Cans by Gc-Ms and Spme. Materials.

[35] Geiss O., Senaldi C., Bianchi I., Lucena A., Tirendi S., Barrero-Moreno J. (2018). A Fast and Selective Method for the Determination of 8 Carcinogenic Polycyclic Aromatic Hydrocarbons in Rubber and Plastic Materials. J. Chromatogr. A.

[36] Arce M.M., Sanllorente S., Ortiz M.C., Sarabia L.A. (2018). Easy-to-Use Procedure to Optimise a Chromatographic Method. Application in the Determination of Bisphenol-a and Phenol in Toys by Means of Liquid Chromatography with Fluorescence Detection. J. Chromatogr. A.

[37] Sung J.H., Kwak I.S., Park S.K., Kim H.I., Lim H.S., Park H.J., Kim S.H. (2010). Liquid Chromatography-Tandem Mass Spectrometry Determination of N-Nitrosamines Released from Rubber or Elastomer Teats and Soothers. Food Addit. Contam. Part A Chem. Anal. Control Expo. Risk Assess..

[38] Sternbauer L., Dieplinger J., Buchberger W., Marosits E. (2014). Determination of Nucleating Agents in Plastic Materials by Gc/Ms After Microwave-Assisted Extraction within Situ Microwave-Assisted Derivatization. Talanta.

[39] Gimeno P., Thomas S., Bousquet C., Maggio A., Civade C., Brenier C., Bonnet P. (2014). Identification and Quantification of 14 Phthalates and 5 Non-Phthalate Plasticizers in Pvc Medical Devices by Gc–Ms. J. Chromatogr. B.

[40] Garrigós M.C., Marín M.L., Cantó A., Sánchez A. (2004). Determination of Residual Styrene Monomer in Polystyrene Granules by Gas Chromatography–Mass Spectrometry. J. Chromatogr. A.

[41] Dong C., Liu Y., Yang W., Sun X., Wang G. (2013). Simultaneous Determination of Phthalate Plasticizers in Pvc Packaging Materials Using Homogeneous-Ultrasonic Extraction-Gc-Ms Assisted with Continuous Wavelet Transform. Anal. Methods.

[42] Liang Z.H., Luo Q., Fan H.B., Lv S.H., Zeng Y.Y. (2013). Determination of Phthalate Esters in the Children’s Products Using Ultrasonic Extraction and Gas Chromatography. Adv. Mater. Res..

[43] Gawlik-Jędrysiak M. (2013). Determination of Phthalate Esters Content in Plastic Articles: Comparison of Extraction Methods. J. Anal. Chem..

[44] Pöhlein M., Bertran R.U., Wolf M., van Eldik R. (2008). Versatile and Fast Gas Chromatographic Determination of Frequently Used Brominated Flame Retardants in Styrenic Polymers. J. Chromatogr. A.

[45] Wang Q., Storm B.K. (2005). Separation and Analysis of Low Molecular Weight Plasticizers in Poly(Vinyl Chloride) Tubes. Polym. Test..

[46] Shen H. (2005). Simultaneous Screening and Determination Eight Phthalates in Plastic Products for Food Use by Sonication-Assisted Extraction/Gc–Ms Methods. Talanta.

[47] Lv Q., Zhang Q., Li W., Li H., Li P., Ma Q., Meng X., Qi M., Bai H. (2013). Determination of 48 Fragrance Allergens in Toys Using Gc with Ion Trap Ms/Ms. J. Sep. Sci..

[48] Meng X., Zhang N., Sun X., Niu Z., Deng Y., Xu J., Bai H., Ma Q. (2020). Suspect Screening of 200 Hazardous Substances in Plastic Toys Using Ultra-High-Performance Liquid Chromatography-Hybrid Quadrupole Time-of-Flight Mass Spectrometry. J. Chromatogr. A.

[49] Hajšlová J., Holadová K., Kocourek V., Poustka J., Godula M., Cuhra P., Kempný M. (1998). Matrix-Induced Effects: A Critical Point in the Gas Chromatographic Analysis of Pesticide Residues. J. Chromatogr. A.

[50] Rahman M.M., Abd El-Aty A.M., Shim J. (2013). Matrix Enhancement Effect: A Blessing Or a Curse for Gas Chromatography?—A Review. Anal. Chim. Acta.

[51] Williams M.L., Olomukoro A.A., Emmons R.V., Godage N.H., Gionfriddo E. (2023). Matrix Effects Demystified: Strategies for Resolving Challenges in Analytical Separations of Complex Samples. J. Sep. Sci..

